# The First Use of the Global Oral Cholera Vaccine Emergency Stockpile: Lessons from South Sudan

**DOI:** 10.1371/journal.pmed.1001901

**Published:** 2015-11-17

**Authors:** Abdinasir Abubakar, Andrew S. Azman, John Rumunu, Iza Ciglenecki, Trina Helderman, Haley West, Justin Lessler, David A. Sack, Stephen Martin, William Perea, Dominique Legros, Francisco J. Luquero

**Affiliations:** 1 World Health Organization, Juba, Republic of South Sudan; 2 Johns Hopkins Bloomberg School of Public Health, Baltimore, Maryland, United States of America; 3 Ministry of Health, Juba, Republic of South Sudan; 4 Médecins Sans Frontières, Geneva, Switzerland; 5 Medair, Ecublens, Switzerland; 6 International Organization for Migration, Juba, Republic of South Sudan; 7 World Health Organization, Geneva, Switzerland; 8 Epicentre, Paris, France

## Abstract

Andrew Azman and colleagues describe their experience of deploying >250,000 doses of oral cholera vaccine in South Sudan in 2014

**Summary Points**

- A global oral cholera vaccine (OCV) stockpile was established in 2013 to improve rapid access to the vaccine in outbreaks and emergencies in which cholera risk is high. The first deployment from the global OCV stockpile was to South Sudan in 2014 because of high cholera risk from massive population displacements within the civil war.
- 256,700 doses of OCV were delivered, with high coverage, throughout the country as part of a comprehensive cholera prevention strategy by multiple agencies, some of which had little to no previous experience with this vaccine.
- A cholera epidemic began during vaccination, and a basic comparison of epidemic curves in vaccinated and unvaccinated areas suggests little to no transmission occurred in vaccinated areas, though more in depth analysis is needed.
- This deployment highlights the feasibility of effective deployments from the OCV stockpile and the importance of strong coordination between governmental and nongovernmental agencies in cholera prevention and control planning from the assessment of cholera risk to the deployment of the vaccines.
- A larger global supply of OCV is urgently needed to cover those most in need. With limited vaccine availability now and likely in the upcoming years, more work is needed on deciding how to most efficiently use the vaccine, which may include alternative dosing regimens and targeting specific subpopulations.

## Background

In December 2013, violence erupted in South Sudan’s capital, Juba, and quickly spread throughout the country. The crisis exacerbated an already dire humanitarian situation in the youngest and one of the poorest countries in the world, where 40% of the population had access to basic health services, 70% to an improved water supply, and only 13% to adequate sanitation facilities [1,2]. Continued fighting and the threat of escalating violence resulted in displacement of more than one in five people throughout the country, many of them residing in Protection of Civilian (PoC) sites inside United Nations Mission bases and spontaneous settlements of internally displaced persons (IDPs).

## The Decision to Use Oral Cholera Vaccine (OCV)

The appalling conditions of the PoC and IDP camps throughout the country led to early discussions between the South Sudan Ministry of Health (MoH), the World Health Organization (WHO), and other partners about the possibility of a cholera outbreak exploding within the camps. Based on the perceived success of a preventative cholera vaccination campaign in Maban County, South Sudan, the previous year [3], the MoH and partners agreed that oral cholera vaccination should be part of an emergency preventative health package and commissioned a rapid risk assessment. The assessment, conducted by the MoH and WHO in January 2014, confirmed that the poor water and sanitation conditions, poor hygiene behaviors, low nutritional status of IDPs, overcrowded camps, and high acute watery diarrhea rates all pointed to an increased likelihood of a cholera epidemic with significant morbidity and mortality if cholera were to be introduced to the camps.

**Fig 1:**
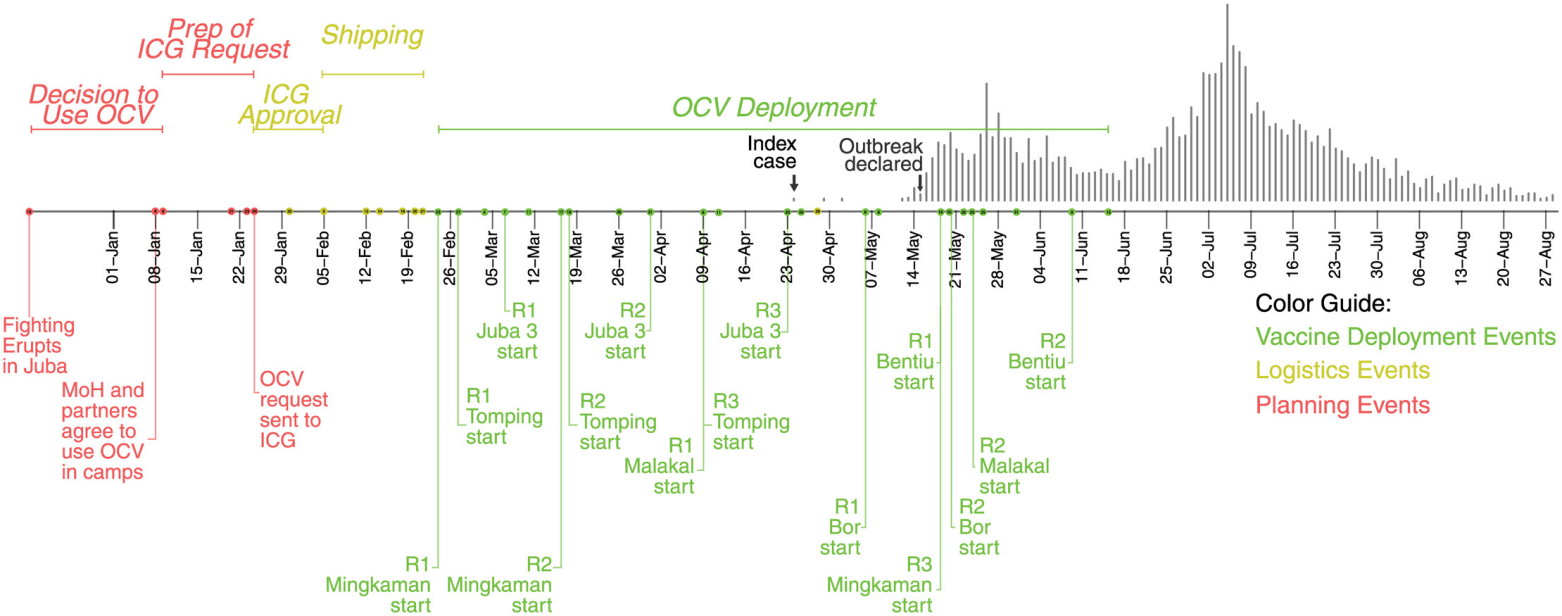
Timeline of key vaccination events in South Sudan in 2014. The dots on the timeline represent key vaccination-related events throughout the country. Labels are shown for a selection of events, with the complete timeline shown in S1 Fig. Colors represent different phases of the vaccination campaigns, with red representing planning events, yellow representing logistical activities leading up to the vaccine deployment, and green representing vaccination deployment activities. The epidemic curve is shown as grey vertical bars above.

Within six weeks from the start of the emergency (Fig 1, S1 Fig), the MoH made the first-ever request to the International Coordinating Group (ICG) to access the global OCV stockpile (http://www.who.int/cholera/vaccines/ocv_stockpile_2013/en/) while at the same time planning for increased water sanitation and hygiene activities through the National Cholera Taskforce. The emergency stockpile, established in 2013, currently manages most of the world’s (limited) supply of Shanchol (Shantha Biotechnics, a Sanofi Company), one of the two internationally licensed OCVs, with 2–3 million doses available in 2014 and 2015 and more projected for future years. This stockpile follows a similar model to other existing emergency vaccine stockpiles like those for yellow fever and meningitis to allocate vaccines rapidly for outbreaks and other situations in which disease risk is high [4].

The target population for this first request included 163,000 IDPs but did not include the broader host communities because of limited vaccine availability and the assessed lower risk of spread in these areas. Within one week, part of the request (188,000 doses) was approved with a second approval for other locations (including Juba) provided a week after (64,000 doses). The first batch of OCV arrived in country 30 days after the initial request was made and 24 days after initial approval because of shipping and logistical constraints (Fig 1, S1 Fig).

## Multisite Multipartner Oral Cholera Vaccination Campaigns

**Fig 2:**
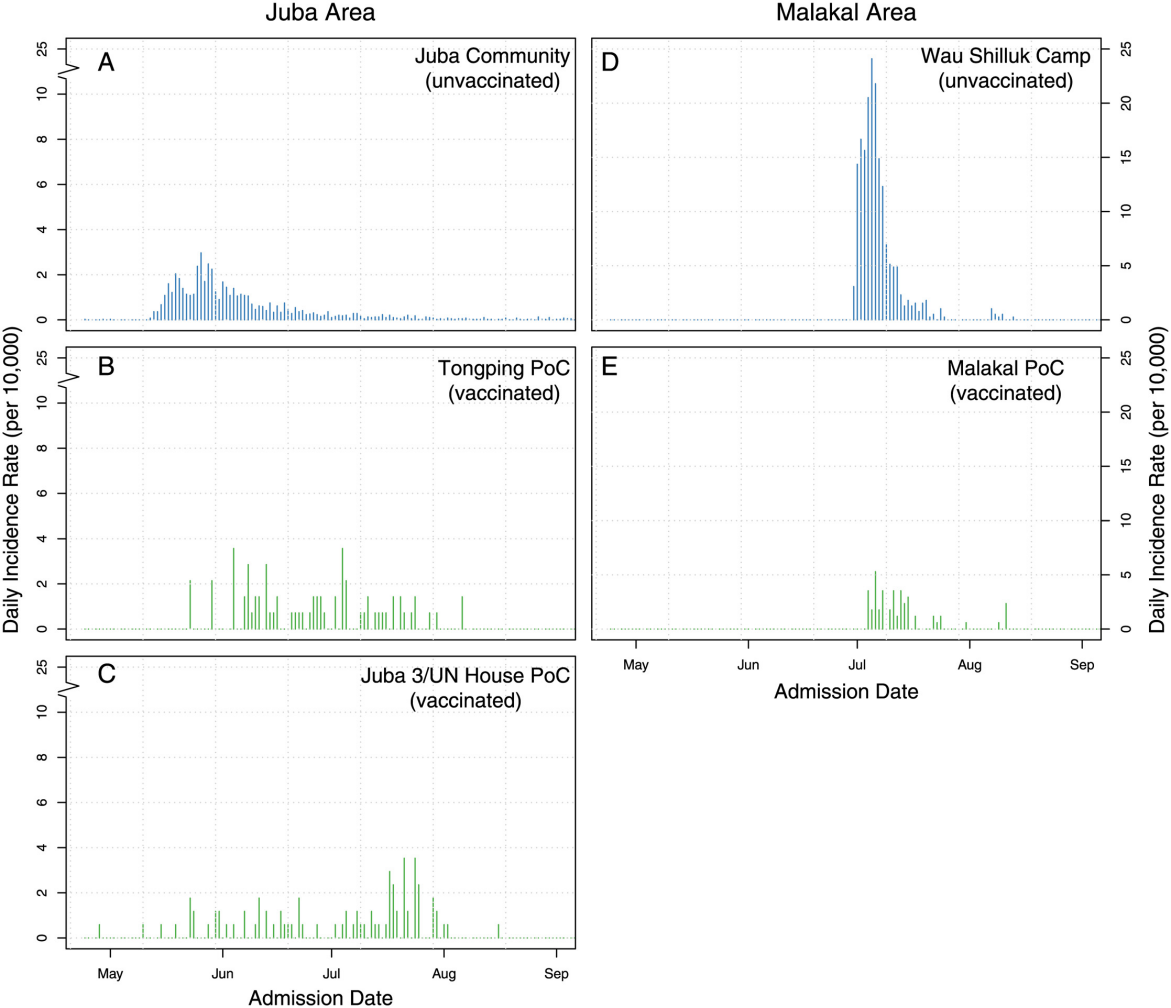
Epidemic curves of suspected cholera cases in key vaccinated (green) and unvaccinated areas (blue). Panels show the daily incidence rate of suspected cholera cases in the Juba community (A), the Juba PoC camps (B–C), Wau Shilluk camp (D), and Malakal PoC camp (E). Note that panels A–C have a break in the *y*-axis, as they had a much smaller incidence rate.

This intervention targeted all nonpregnant IDPs who were at least one year old in PoC camps in Juba (Juba 3 and Tongping), Bentiu, Bor, and Malakal, in addition to Mingkaman camp (Fig 2). Unlike other OCV campaigns [3,5,6], this first deployment from the stockpile involved multiple agencies with shared roles and responsibilities. The WHO coordinated the vaccine request and the overall strategy and ensured coherence among agencies participating in the immunization sessions. United Nations Children's Fund (UNICEF) kept the central stock of vaccine and guaranteed storage under adequate cold chain. The campaigns were implemented by four different agencies: Médecins Sans Frontières (MSF), Medair, International Organization for Migration, and International Medical Corps. Only MSF had prior experience leading an OCV campaign.

The first campaign started on 23 February in Mingkaman, and the last one on 19 May in Bentiu; thus, nearly continuous vaccination activities occurred in different parts of the country for three months (Fig 1). In three of the six sites, an additional (third) vaccination round was conducted to provide an additional opportunity for new arrivals to receive the second and final dose of OCV (Table 1). Ultimately, 256,700 doses were delivered through fixed vaccination posts and mobile vaccination teams, with interdose timing ranging from 12 to 56 days (Table 1, S1 Table, Fig 1, and S1 Fig).

**Table 1:** Details of OCV deployments in South Sudan in 2014 by site.

Location	County	State	Target Population^‡^	Vaccine Rounds	Doses Used	Average Daily Doses Used	First Dose Vaccine Coverage	Second Dose Vaccine Coverage	Coverage Assessment Method
Tongping Camp	Juba	Central Equatoria	16,936	3	28,942	2,205	94%	93%	LQAS
UN House Camp	Juba	Central Equatoria	11,640	3	14,315	1,143	96%	95%	LQAS
Mingkaman Camp	Awerial	Lakes	84,000	3	110,997	4,294	82%	64%	Cluster Sample Survey
Bor Camp	Bor	Jonglei	3,273	2	5,809	1,452	92%	86%	Administrative Coverage
Bentiu Camp	Rubkona	Unity	28,800	2	66,529	5,544	*	*	
Malakal Camp	Malakal	Upper Nile	17,928	2	30,108	7,527	97%	92%	Coverage Survey
*Total*			*162*,*577*		*256*,*700*				

LQAS, lot quality assurance sampling

^**‡**^ Target population estimates were made at the time of requesting vaccine.

*The campaign was conducted during a large population influx, so no reliable denominator figures were available.

Vaccine coverage was estimated using different methods between locations (S1 Table). Lot quality assurance sampling (LQAS, [7]) was conducted in Juba camps, and a cluster-based household survey was conducted in Mingkaman; in the other sites, only administrative vaccine coverage was available (i.e., doses delivered/target population size). First dose coverage estimates ranged from 82% in Mingkaman to 97% in Malakal, with second-dose coverage estimates ranging from 64% (Mingkaman) to 95% (Juba, UN House) (Table 1, S1 Table). Though the coverage estimates were generally high, each comes with a different degree of uncertainty because of the methods used; thus, comparisons should be made with caution.

Although costing data was not systematically collected from each agency involved, these campaigns allow for some new insight into the costs of vaccine delivery and quality control. From two agencies (MSF and Medair) that delivered the majority of the vaccines, the delivery cost per vaccine was US$0.63 and US$0.73 (excluding international staff). While these costs can likely be reduced as agencies gain more experience with OCV, the cost of each dose (currently US$1.85 through the stockpile) will likely continue to dominate campaign costs in the near term.

## Cholera Strikes South Sudan

**Fig 3:**
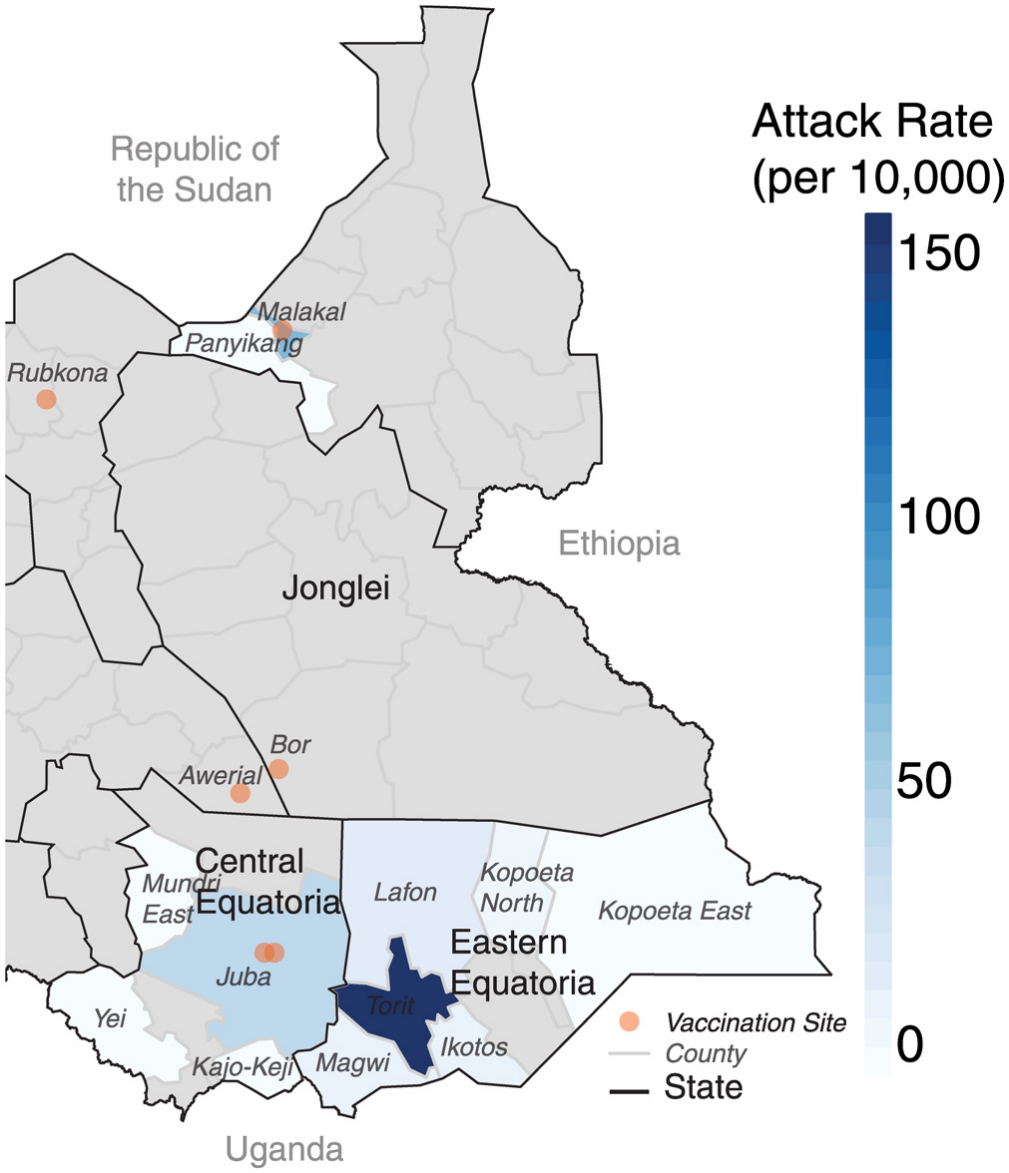
Illustration of the county-level attack rates in the 2014 cholera epidemic with respect to the vaccination locations (orange dots). Grey areas represent counties with no suspected cholera cases.

After a period of five years without confirmed cholera in South Sudan, a suspected cholera case was reported from UN House/Juba 3 camp in Juba on 29 April (approximately two months from the start of vaccination activities, Fig 1). Although the origins of this reported infection are unknown, a cholera epidemic had been reported in a neighboring area of Uganda the previous week [8]. Following field investigations and laboratory confirmation, on 15 May, the MoH declared a cholera outbreak in Juba caused by *Vibrio cholerae* O1 Inaba sensitive to tetracycline and ciprofloxacin. By the end of October, 6,269 suspected cases had been reported throughout the country (Fig 3), including 105 deaths in health facilities and 51 community deaths (case fatality ratio 2.4%).

From Juba and its surrounding areas, where most cases occurred outside of the camps, the epidemic spread east to Torit (Eastern Equatoria) in June (Fig 3). In Eastern Equatoria, the slow outbreak response and relatively poor baseline health care infrastructure led to high case fatality ratios around the state, reaching as high as 11% (21 deaths) in Ikotos county. The epidemic then jumped over 1,000 kilometers north (presumably from an infected traveler), over a large area with no confirmed cholera, to the states of Upper Nile and Jonglei, where it almost exclusively affected displaced persons living outside the vaccinated PoC camps. Of the five areas vaccinated countrywide, only two (the Malakal and Juba PoC camps) experienced cases either within the population targeted for vaccination or the surrounding area (i.e., the host community or nearby informal settlements).

Some cholera cases were reported in vaccinated camps, but there was little indication of transmission. The epidemic curves in the vaccinated camps did not have the typical epidemic curve observed during cholera outbreaks (i.e., only sporadic cases reported without a clear peak followed by a decline, Fig 2B, 2C and 2E). In comparison, a more typical epidemic curve, suggestive of transmission, was observed in the unvaccinated surrounding areas (Fig 2A and 2D). This is especially striking in the comparison of Malakal and Wau Shilluck, two relatively similar camps just across a river from one another. Despite the suggestive visual evidence that vaccination slowed transmission in the vaccinated areas, more rigorous analyses are needed (analyses progress at the time of writing this manuscript) to assess the full impact of this vaccination campaign on the population.

## Lessons Learned

This first deployment of OCV through the global stockpile provided ~250,000 doses of OCV to high-risk populations months before the onset of a cholera epidemic within the country. Strong leadership by the MoH and WHO, pre-existing coordination structures (e.g., the UN Health Cluster), clearly defined roles and responsibilities of the different actors (i.e., one lead agency per site), and previous use of the vaccine played crucial roles in ensuring the success of these campaigns during a humanitarian emergency. This experience involved non-medically focused agencies without prior OCV experience, which points towards the possibility of diversifying the actors involved in future campaigns in order to avoid missed opportunities for OCV use and to improve its integration into cholera control and prevention programs.

Despite the preventative vaccination campaign, it was not enough to prevent an outbreak within the country, and several issues prevented additional reactive use of OCV. The competing priorities for finite (financial and human) resources in complex emergencies are exacerbated during outbreaks, and difficult decisions must be made through weighing the costs and benefits of different interventions. When the cholera outbreak was declared in Juba, an analysis of (limited) historical data and a risk assessment conducted early on in the epidemic pointed to some unvaccinated areas at high risk for cholera introduction and spread (e.g., Torit and Wau Shilluk). However, despite these warnings, additional vaccination was not used because of limited resources within South Sudan and the global stockpile and limited access to key populations. Even with interested policy makers, it was difficult to motivate operational organizations to engage in reactive vaccination, as they were consumed with other outbreak response activities, though the spatiotemporal pattern of spread within this epidemic suggests that reactive vaccination in many of these (at the time) unaffected high-risk areas surrounding the outbreak may have been an efficient mechanism to limit its spread.

The vaccines arrived in South Sudan about 30 days after the request to the stockpile. In this preemptive deployment, the delay likely had no impact on the overall vaccine-attributable reductions in cholera cases once the epidemic started. However, in other settings, particularly where OCV is being used to fight an ongoing epidemic, a quicker timeline from submission of the vaccine request to delivery in country is crucial. While some delays are inevitable and out of the control of the ICG, lessons from this and future stockpile deployments should be used to improve the lead times between the submission of the ICG request and the delivery of the vaccines.

The limited global supply of OCV made it difficult to decide on the target population for reactive campaigns and to advocate for an expanded preemptive target population. If more doses would have been available, it is possible that the host communities, including Juba, where over 2,000 cases and 40 deaths were reported, and informal IDP settlements would have received OCV. The WHO cholera stockpile was designed to have 2 million doses (subject to global production constraints) for outbreak response and emergencies, which if fully available to a single epidemic, would often eliminate challenges in deciding what population to target. Nonetheless, the imbalance between the number of doses available and the population at risk (2.8 billion [9]) means that difficult choices will likely be confronted. Thus, tools to identify populations in which the health benefits might be maximized in a timely manner (e.g., populations with the highest mortality risk or disease risk) are a clear priority that could facilitate the OCV decision-making process.

Despite the fact that many (still) displaced people are today protected by vaccine, the water sanitation and hygiene conditions in the camps remain dismal and conducive to the spread of cholera and/or other waterborne diseases. With the continued population movements and a high birth rate, vaccine alone will likely not protect the entire population from another outbreak. Investments in vaccine must be coupled with long-term commitments to improving water sanitation and hygiene conditions if sustainable gains in cholera-attributable morbidity and mortality are to be made.

## Way Forward

This first deployment of OCV through the global stockpile highlighted how this mechanism can be efficiently used to protect individuals and slow the transmission of cholera, even in complex emergencies.

More work is needed in understanding how to target appropriate populations if OCV is to become more integrated into standard cholera control and prevention packages. With enhancement of cholera surveillance systems and analyses of historical epidemiologic data, target populations may be identified more precisely—whether based on geography [10], age [11], or other criteria [12]. More evidence is needed on alternative dosing strategies, and field-adapted variants of currently licensed OCV should be fast-tracked. Changes to the ways in which the current vaccine is administered and/or development of better vaccines could greatly increase the feasibility of OCV campaigns, including changes to the packaging, reduction in the cold-chain requirements (i.e., a controlled temperature chain strategy), and the possibility of using a reduced one-dose regimen [13]. Above all, the dissemination of the evidence supporting OCV use in cholera epidemics will be important for its adoption by countries and humanitarian actors alike. The global OCV stockpile is an asset to the public health community, and we must continue to improve awareness of the vaccine as an outbreak prevention and control tool. At the same time, we must seek smarter deployment strategies and the development of better vaccines in order to maximize the benefits and minimize the costs.

## Supporting Information

S1 Fig Timeline of key vaccination events in South Sudan in 2014 with additional details. (EPS)

S2 Fig Number of suspected cases per day in the three counties with the most cases during the epidemic (top three panels) and all of the other counties on the bottom panel. (EPS)

S1 Table Details of OCV campaigns throughout South Sudan in 2014. (DOCX)

## Abbreviations

ICG: International Coordinating Group
IDP: internally displaced person
LQAS: lot quality assurance sampling
MoH: Ministry of Health
MSF: Médecins Sans Frontières
OCV: oral cholera vaccine
PoC: Protection of Civilians
UNICEF: United Nations Children's Fund
WHO: World Health Organization
