# Supplementary material for: The First Use of the Global Oral Cholera Vaccine Emergency Stockpile: Lessons from South Sudan
Source: PLoS Med. 2015 Nov 17;12(11):e1001901. doi: 10.1371/journal.pmed.1001901 (PMC4648513; doi:10.1371/journal.pmed.1001901)
Supplement: S1 Table — (DOCX) [file pmed.1001901.s003.docx]

| **Location** | **County** | **State** | **Target Pop.** | **Start Day Round 1** | **End Day Round 1** | **Doses Delivered Round 1** | **Start Day Round 2** | **End Day Round 2** | **Doses Delivered Round 2** | **Start Day Round 3** | **End Day Round 3** | **Doses Delivered Round 3** | **Total Doses Delivered** | **Avg. Doses per Day** | **First Dose Vaccine Coverage** | **Second Dose Vaccine Coverage** | **Coverage Assessment Methodology** | **Implementing Organization** |
| --- | --- | --- | --- | --- | --- | --- | --- | --- | --- | --- | --- | --- | --- | --- | --- | --- | --- | --- |
| Tomping PoC | Juba | Central  Equatoria | 16,936 | 27-Feb | 4-Mar | 14,112 | 18-Mar | 26-Mar | 12,695 | 9-Apr | 11-Apr | 2,135 | 26,807 | 2,205 | 94% | 93% | LQAS | Medair |
| UN House PoC | Juba | Central  Equatoria | 11,640 | 7-Mar | 11-Mar | 6,920 | 31-Mar | 11-Apr | 6,123 | 23-Apr | 25-Apr | 1,272 | 13,043 | 1,143 | 96% | 95% | LQAS | Medair |
| Mingkaman PoC | Awerial | Lakes | 84,000 | 23-Feb | 4-Mar | 48,906 | 17-Mar | 24-Mar | 38,196 | 19-May | 31-May | 23,895 | 87,102 | 4,294 | 82% | 64% | Cluster Survey | MSF |
| Bor PoC | Bor | Jongeli | 3,273 | 6-May | 8-May | 2,862 | 20-May | 22-May | 2,947 | - | - | - | 5,809 | 1,452 | 92% | 86% | Administrative Coverage | IOM |
| Rubkona IDP Camp | Bentiu | Unity | 28,800 | 19-May | 25-May | 34,370 | 9-Jun | 15-Jun | 32,159 | - | - | - | 66,529 | 5,544 | * | * | ¶ | IOM/UNICEF |
| Malakal PoC | Malakal | Upper Nile | 17,928 | 9-Apr | 11-Apr | 16,055 | 23-May | 25-May | 14,053 | - | - | - | 30,108 | 7,527 | 97% | 92% | Survey | MSF/IOM/UNICEF |

¶Campaign conducted during large population influx so no reliable denominator figures
